# Non-coding RNAs participate in interactions between senescence and gastrointestinal cancers

**DOI:** 10.3389/fgene.2024.1461404

**Published:** 2025-01-03

**Authors:** Zhao-Zhe Liu, Fa-He Ji, Ying Piao

**Affiliations:** Department of Oncology, General Hospital of Northern Theater Command, Shenyang, China

**Keywords:** non-coding RNAs, senescence, gastrointestinal cancers, miRNAs, lncRNAs, CircRNAs

## Abstract

Relationships between cellular senescence and gastrointestinal cancers have gained prominence in recent years. The currently accepted theory suggests that cellular senescence and cancer occurrence exhibit “double-edged sword” effects. Cellular senescence is related to cancer via four “meta-hallmarks” i.e., genomic instability, epigenetic alterations, chronic inflammation, and dysbiosis, along with two “antagonistic hallmarks” i.e., telomere attrition and stem cell exhaustion. These relationships are characterized by both agonistic and antagonistic elements, but the existence of an intricate dynamic balance remains unknown. Non-coding RNAs (ncRNAs) have vital roles in post-transcriptional regulation, but how they participate in agonistic and antagonistic relationships between cellular senescence and gastrointestinal cancers remains to be fully investigated. In this article, we systematically review how ncRNAs (including microRNAs (miRNAs), long ncRNAs (lncRNAs), and circularRNAs (circRNAs)) participate in interactions between cellular senescence and gastrointestinal cancers. Our aim is to elucidate a triangular relationship between “ncRNAs–senescence–gastrointestinal cancers” which considered these three elements as an equal important standing. We are keen to identify prognostic or therapeutic targets for gastrointestinal cancers from, i.e., aging-related ncRNAs, or discover novel strategies to treat and manage in the elderly. We seek to clarify complex relationships where ncRNAs participate in “senescence–gastrointestinal cancers” interactions.

## 1 Background

Approximately 20 years ago, non-coding RNAs (ncRNAs), including microRNAs (miRNAs), long ncRNAs (lncRNAs), circular RNAs (circRNAs), tRNA-derived stress-induced RNAs (tiRNAs), and PIWI-interacting RNAs (piRNAs) were considered as genetic “junk”, seemingly devoid of use even when spliced out (Yan and Bu, 2021). Since then, many studies have revealed that ncRNAs are functional single-stranded RNA molecules (Tiwari et al., 2018; Li et al., 2020). Due to their abundance in gene expression products, they often form network-like connections during transcriptional regulation via endogenous competition (Anastasiadou et al., 2018). Long-chain molecules such as lncRNAs and circRNAs competitively bind to short-chain molecules like miRNAs, interfering with coding gene transcription. Additionally, long single-stranded RNA molecules such as lncRNAs also function as “pseudogenes”, assuming gene-like roles (Slack and Chinnaiyan, 2019).

Cellular senescence, a multifaceted expression of physiological deterioration and malfunction during an organism’s degenerative phase, signifies the progressive reduction in cell proliferation, differentiation abilities, and physiological functions as cells engage in life-sustaining activities over time. The molecular signatures of cellular senescence encompass various phenomena: alterations in cell morphology (such as flattening, enlargement, and vacuolation), cell cycle arrest, nuclear changes (including global heterochromatin loss and localized increases), the senescence-associated secretory phenotype (SASP), specific cell surface markers (like DPP4 and TNFRSF10D), elevated lysosomal content, metabolic adaptations, genomic instability marked by histone variant γH2AX phosphorylation, epigenetic modifications (such as DNA hypomethylation and non-coding RNA regulation), telomere shortening, and mitochondrial dysfunction. Research has identified numerous well-known signaling pathways as being associated with senescence, including the Insulin/IGF-1 signaling (IIS) pathway, the mTOR pathway, the AMPK pathway, the NF-κB pathway, and the Sirtuins pathway (McHugh et al., 2024; Schmitt et al., 2022).

Cellular senescence represents inevitable natural stages as life reaches its end (Di Micco et al., 2021; Calcinotto et al., 2019). Many studies now indicate that aging serves as a risk factor promoting cancer occurrence and development (Harris et al., 2014; Pandics et al., 2023). Intriguingly, a phenomenon exists whereby cancer incidences peak and subsequently decline; this apex is reached at age 85, and declines after 90. Beyond the age of 100, the likelihood of cancer-related mortality drops below 5%. López-Otín et al. in their 2023 study in Cell Metabolism, reported that cellular senescence was associated with cancer via four “meta-hallmarks” i.e., genomic instability, epigenetic alterations, chronic inflammation, and dysbiosis, and two “antagonistic hallmarks” i.e., telomere attrition and stem cell exhaustion (Lopez-Otin et al., 2023). Interactions between cellular senescence and cancer exhibit both agonistic and antagonistic features, with an unknown balance point in the intricate relationship. Furthermore, aging exhibits two complex characteristics–giant autophagy dysfunction and cellular senescence–with dual roles inhibiting and promoting cancer. Of the four “meta-hallmarks”, the second characteristic, epigenetic alterations, include changes in DNA methylation, histone modification, chromatin remodeling, and ncRNAs.

In López-Otín et al.’s report, ncRNAs are briefly presented as one aspect of a meta-hallmark, where authors provide only two examples: the cancer-inhibiting miR-455-3p and phosphorylation changes in the Dicer enzyme (Lopez-Otin et al., 2023). However, throughout the review, the authors consistently assert that any meta-hallmarks, including ncRNAs, synergistically contribute to senescence and cancers rather than acting as third parties with opposing effects on senescence and cancers. Firstly, miR-455-3p is a cancer-inhibiting miRNA; its overexpression, proven to extend lifespan by protecting neural function (Kumar et al., 2021), also inhibits hepatocellular cancer (HCC) growth (Ma et al., 2022), as inhibiting HCC development is associated with increased lifespan. Conversely, reducing miR-455-3p in mice causes cognitive impairment and a shortened lifespan (Kumar et al., 2021). However, its downregulation in human cancer cells promotes osteosarcoma cell proliferation and invasion (Yi et al., 2020). Therefore, based on conclusions from these three studies, the authors suggest a causal relationship between this miRNA, senescence, and cancer because it inhibits HCC growth and extends lifespan, and lifespan extension itself has anti-aging effects. Another example is DICER1, a key miRNA cleavage enzyme. When its phosphorylation output is mutated, miRNA formation is impeded. Aryal et al., in their two 2019 studies, observed that mutated DICER1 phosphorylation in mouse models altered metabolism-associated miRNAs, induced a hypermetabolic phenotype, and accelerated aging (Aryal et al., 2019a; Aryal et al., 2019b). Simultaneously, the mutated phosphorylation status caused mice to develop lung cancer or other tumors via KRAS and P53 pathways, thus promoting cancer development (Aryal et al., 2019b), although this latter study did not refer to miRNA research. Therefore, these authors did not directly elucidate a triangular relationship between the three factors: “ncRNAs–senescence–gastrointestinal cancer”.

**FIGURE 1:**
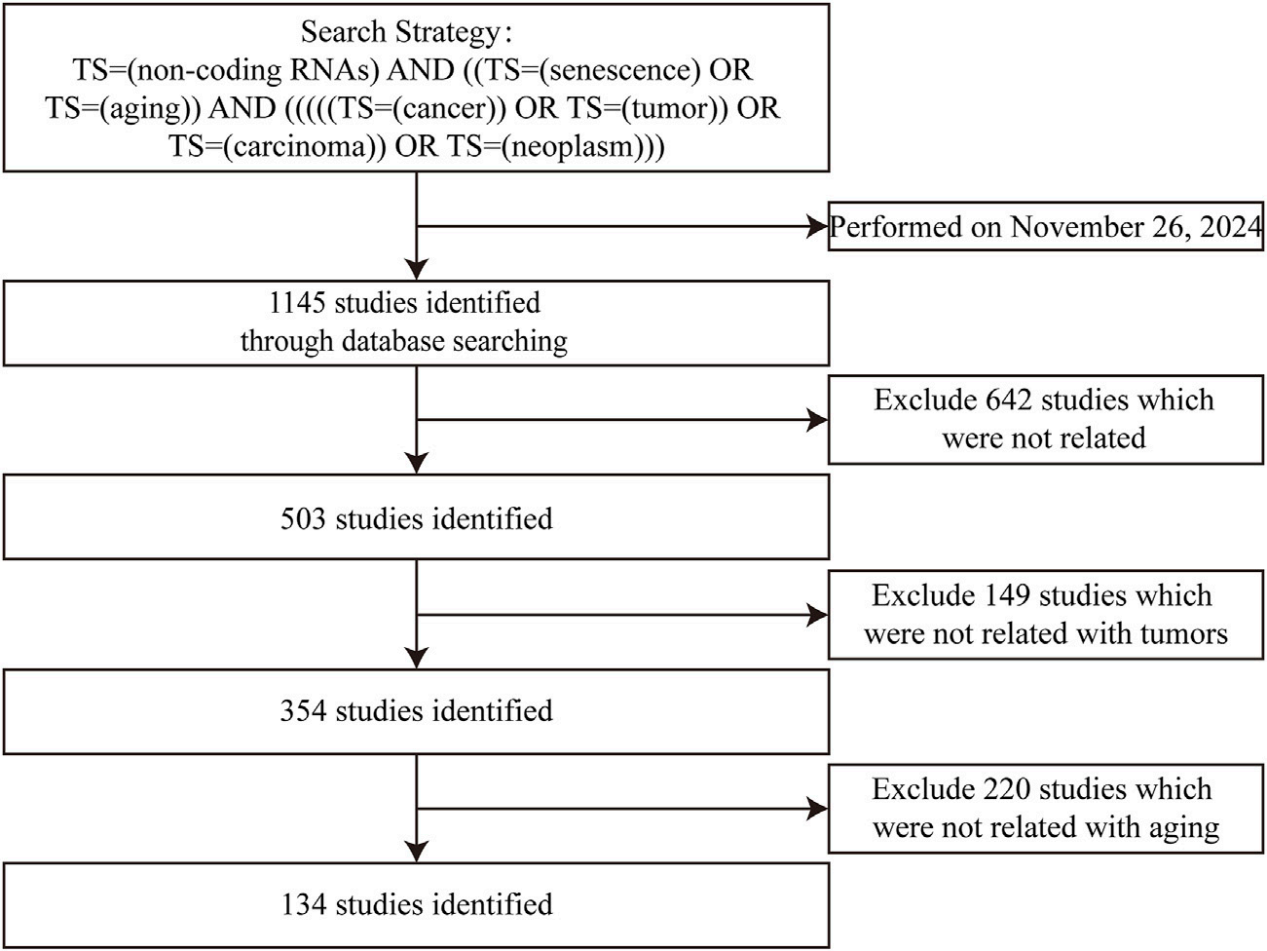
Studies identified with criteria for exclusion for this systemic review.

Current reviews often separately discuss “ncRNAs–senescence” and “ncRNAs–cancers,” treating them as parallel relationships without exploring their interconnectedness. In this study, we systematically review ncRNAs, including miRNAs, lncRNAs, circRNAs and tRNAs, which participate in interactions between cellular senescence and gastrointestinal cancers. The flowchart for screening articles for this system review is shown in Figure 1. Our aim is to clarify the “triangular” relationship between “ncRNAs–senescence–gastrointestinal cancers”, with a view to identifying prognostic or therapeutic aging-related ncRNA targets for gastrointestinal cancers or discovering new strategies to treat and manage gastrointestinal cancers in the elderly. Our remit is to clarify complex relationships where ncRNAs participate in “senescence–gastrointestinal cancers” interactions.

## 2 MiRNAs

In contemporary research investigating correlations between senescence and gastrointestinal cancers, miRNAs are the most extensively studied ncRNAs when compared to lncRNAs and circRNAs. This research encompasses many cancers, including esophageal, gastric, colorectal, liver, and pancreatic cancer (Table 1). Primarily focusing on miRNAs, most studies have explored their expression, biological effects, and potential pathogenic mechanisms, but direct research addressing miRNA contributions to senescence–gastrointestinal cancers relationships remain limited at mechanistic levels. The majority of studies view miRNAs as implicated in potential carcinogenic mechanisms, with few delving into potential aging mechanisms. Additionally, a minority of studies have focused on coding gene roles in “senescence–gastrointestinal cancers” relationships, where miRNAs are identified only as target molecules to that coding gene.

**TABLE 1:** The characteristics of the miRNAs participating in interactions between senescence and gastrointestinal cancers.

Model type	Species	NcRNAs	Expression	Biological functions	Mechanism	Reference
CSCs in PDA	Human	MiR-17-92 cluster	Downregulated in chemoresistant CSCs versus non-CSCs	Increased CSC proliferation of PDA	Targeted multiple NODAL/ACTIVIN/TGF-β1 signaling cascade members as well as directly inhibited the downstream targets p21, p57 and TBX3	Cioffi et al. (2015)
AMSCs	Mice	MiR-17-92 cluster	MiR-17 and miR-20a was obviously decreased in oAMSCs and replicatively senescent AMSCs	oAMSCs exhibited senescence phenotypes	C-Myc-regulated miR-17-92 contributed to increased p21 expression and redox system dysregulation during AMSC senescence	Cen et al. (2023)
CSLCs in CRC	Rat	MiR-21	Upregulated	Aging is also accompanied by increased expression of miR-21 in colon	Inhibition of EGFR using the EGFR inhibitor cetuximab abrogated the age-related increase in CD166 and ALDH-1 as well as miRNA miR-21	Nautiyal et al. (2012)
CRC	Human	MiR-22-3p	Downregulated in TCGA and GEO databases	Inhibited cell senescence	AP4, miR-22-3p and MDC1 form a conserved and coherent, regulatory feed-forward loop to promote DNA repair, which suppresses DNA damage, senescence and CIN, and contributes to 5-FU resistance	Chou et al. (2022)
ESCC	Human	MiR-34a	Upregulated	The suppression of cell proliferation by miR-34a is mainly relevant to the induction of cellular senescence phenotypes	Downregulation of Sirtuin1 (SIRT1) and upregulation of p53/p21 contributed to the anti-tumor mechanism of miR-34a in wild-type p53 ECa-109 cells	Ye et al. (2016)
GC	Human	MiR-106b-5p	NA	Inhibited cell senescence	Through E2F/miR-106b-5p/p21 axis to modulate the GC cellular senescence	Dong et al. (2018)
ESCC	Human	MiR-125b-5p	Downregulated in ESCC tissues	Induced cell senescence	Through down-regulating the target HMGA2	Mei et al. (2017)
HCC	Human	MiR-125b	Downregulated in HCC tissues	Induced cell senescence	By targeting SIRT6, miR-125b can function as a tumor suppressor to induce the cellular senescence and apoptosis in hepatocellular carcinogenesis	Song et al. (2018)
CRC	Human	MiR-129-3p	NA	Induced cell senescence	MiR-129-3p silenced the Pirh2 protein and led to a significant increase in protein levels of p53 and its downstream target p21, which subsequently induced cell senescence	Fu et al. (2019)
HCC	Human	MiR-145	Downregulated in HCC cell lines	Induced senescence-like G1 arrest in cell cycling	Decrease the targeted stem cell-related Oct4 levels	Jia et al. (2012)
CRC	Human	MiR-183/96/182	Upregulated in TCGA databases	Inhibited cell senescence	β-catenin/miR-183/96/182 cluster/FOXO1 mediated-CRC cellular senescence	Fu et al. (2022)
GC	Human	MiR-200 family	NA	Induced cell senescence	MiR-200 family knockout associated senescence in cancer epithelial cells significantly recruited stromal cells in the tumor microenvironment activated of TGF-β and TNF-α pathways	Yu et al. (2022)
HCC	Human	MiR-212	Downregulated in HCC tissues	Induced cell senescence	Hsa-miR-212-RBP2-CDKI pathway participated in HCC senescence	Liang et al. (2013)
CRC	Human	MiR-339-5p	Downregulated in CRC cell lines	Induced cell senescence	MiR-339-5p/MDM2/P53 mediated apoptosis and senescence in response to CRC cells stress	Zhang et al. (2014b)
CRC	Human	MiR-371b-5p	NA	Inhibited cell senescence	Through modulating the miR-371b-5p/TFAP4 axis induced CRC cellular senescence	Yang et al. (2022)
GC	Human	MiR-449	Be decreased in 1-year-old Gastrin KO mice and in *H. Pylori* infected gastric tissues compared to tissues from wild type animals	Induced cell senescence	Induces senescence and apoptosis by activating the p53 pathway	Bou Kheir et al. (2011)
HCC	Human	MiR-495	Downregulated in HCC tissues and cell lines	Induced cell senescence	MiR-495/CTRP3 axis inhibiting HCC cell growth via arresting cells at the G0/G1 phase/induced senescence	Zhang et al. (2021)
GC	Human	MiR-584-5p	Downregulated in GC tissues and cell lines	Induced cell senescence	MiR-584-5p/WWP1 axis activated the TGFβ signaling pathway	Li et al. (2017b)
CRC	Human	MiR-1827	Downregulated in CRC tissues and cell lines	Induced cell senescence	Through targeted MDM, miR-1827 increases p53 protein levels to increase transcriptional activity of p53 and enhance p53-mediated stress responses, including apoptosis and senescence	Zhang et al. (2016)
HCC	Human	MiR-4510	NA	inhibited HCC cell proliferation and migration and induced senescence	Lowed RAF1 mRNA and protein expression inactivated Wnt and RAS/RAF/MEK/ERK signals	Ghousein et al. (2020)
CRC	Human	MiR-186, miR-216b, miR-337-3p, miR-760	NA	Induced cell senescence	Through the p53-p21(Cip1/WAF1) pathway by protein kinase CKII downregulation-mediated ROS production in HCT116 cells	Kim et al. (2012)
HCC	Human	MiR-139-5p	Downregulated in HCC tissues	Induced cell senescence	EZH2/miR-139-5p/TOP2A axis was in regulating cellular senescence and cell proliferation in HCC	Wang et al. (2023)

Note: PDA, pancreatic ductal adenocarcinoma; AMSCs, adipose tissue-derived mesenchymal stem cells; CSLCs, Cancer stem-like cells; ESCC, esophageal squamous cell cancer; GC, gastric cancer; CRC, colorectal cancer; HCC, hepatocellular cancer; TCGA, the cancer genome atlas; GEO, gene expression omnibus; NA, not available.

### 2.1 Esophageal cancer

#### 2.1.1 MiR-34a

MiR-34a is located at 1p36.22 and is a well-recognized interacting target of p53, with many other targets identified including the conditional senescence-related protein Sirtuin 1(SIRT1) (Hu et al., 2018; Lee et al., 2010). Acknowledged as an aging-related miRNA, miR-34a targets p53 and downstream p21 to form an essential pathway in aging with carcinogenic effects. MiR-34a enhances esophageal squamous cell carcinoma (ESCC) cell proliferation while inducing cell aging. Its pathogenic mechanisms may involve SIRT1 downregulation and the upregulation of the downstream p53/p21 protein pathway (Ye et al., 2016; Iwagami et al., 2018; Park et al., 2020). SIRT1 is a member of the SIRT family of class III histone deacetylases and widely involved in the regulation of cellular senescence (Haigis and Sinclair, 2010; Morris, 2013), suggesting that miR-34a’s anti-tumor mechanisms in ECa-109 cells are executed via SIRT1 and its downstream p53/p21 pathway.

#### 2.1.2 MiR-125b-5p

MiR-125b-5p is located at 19q13.41 and is downregulated in esophagus cancer tissues (Fassan et al., 2013). Mei et al. discovered that miR-125b-5p overexpression inhibited esophagus cell proliferation, migration, and invasion, and also induced cell aging. Simultaneously, miR-125b-5p reduced expression of cell cycle regulatory genes, including CCNA2, CCND1, and CCNE1, and regulated epithelial–mesenchymal–transition (EMT) markers, including E-cadherin, N-cadherin, and the EMT-related transcription factor Slug. Mechanistically, removal of the miR-125b-5p target gene HMGA2 replicated miR-125b-5p overexpression on aforementioned cell cycle regulatory factors and EMT markers (Mei et al., 2017). These observations suggested that miR-125b-5p overexpression partially induced aging by downregulating HMGA2 in ESCC, with HMGA2 recognized as an aging-associated protein (Hammond and Sharpless, 2008; Keane and de Magalhaes, 2013; Yu et al., 2013; Tzatsos and Bardeesy, 2008).

### 2.2 Gastric cancer

#### 2.2.1 MiR-106b-5p

MiR-106b-5p is located at 7q22.1, often presents as miR-106b∼25 clusters (Li F. et al., 2017), and is upregulated in GC at both cellular and tissue levels (Zhang R. et al., 2014; Hou et al., 2015; Wang et al., 2010). However, its expression is downregulated in cellular senescence (Hackl et al., 2010; Daneshpour and Ghadimi-Daresajini, 2023) and recognized targets include p21 (Lindahl et al., 2020; Gibcus et al., 2011). Dong et al. reported that miR-106b-5p overexpression inhibited GC cell aging, while an miR-106b-5p inhibitor induced GC cell aging by upregulating p21 protein expression. Mechanistically, these authors reported that upstream of miR-106b-5p was the transcription factor E2F, which bound to the miR-106b-5p promoter and promoted its transcription. This caused miR-106b-5p binding to the p21 protein and the inhibition of GC cell aging. Thus, the E2F/miR-106b-5p/p21 axis promotes GC cell proliferation by suppressing cellular senescence (Dong et al., 2018) and provides experimental evidence for new GC drug development.

#### 2.2.2 MiR-192/215

MiR-192 is situated on chromosome 11q13.1, while miR-215 resides on 1q41. This family, consisting of miR-192, miR-194, and miR-215, are upregulated in GC, and were labeled as “oncogenes” by Zhang et al. (2018). SET8 was identified as the target protein of miR-192/215 by these authors. SET8 is a SET domain-containing member of the methyltransferase family and catalyzes H4K20me monomethylation. Previous research indicated that miR-192/215/SET8/p53 induced oncogene-induced senescence both *in vivo* and *in vitro* in GC. This mechanism relied on DNA damage responses and p53-promoted senescence pathways, suggesting that miR-192/215-SET8 inhibited GC cell metastasis via p53-senescence signaling (Zhang et al., 2020).

#### 2.2.3 The miR-200 family

The miR-200 family, comprising miR-200a, miR-200b, and miR-200c, is located on chromosomes 1p36.33 and 12p13.31. MiR-200a and miR-200b are both associated with favorable survival in GC (Song et al., 2014). In advanced gastric tumor development stages, miR-200 family members are significantly downregulated, resulting in EMT and increased invasiveness. Yu et al. used a multiple CRISPR/Cas9 system to knock out all miR-200 family members and observed G1/S cell cycle arrest and increased senescence-associated beta-galactosidase (SA-β-Gal) activity in GC cell lines. This observation suggested that the miR-200 family inhibited GC cell senescence. Subsequent analyses identified key senescence-associated secretory phenotype (SASP) components in miR-200 family knockout cells. Both tumor growth factor (TGF)-β and tumor necrosis factor-α pathways were identified as downstream molecules, regulating the senescent state. Yu et al. reported that senescence induced by knocking out the miR-200 family in GC cells helped to significantly recruit stromal cells to the tumor microenvironment. This indicated that the knocked out miR-200 family induced senescence and promoted EMT. Thus, MiR-200 family members may inhibit EMT by suppressing senescence, leading to inhibited tumor invasion and migration (Yu et al., 2022).

#### 2.2.4 MiR-449

MiR-449 is situated on 5q11.2 and is downregulated in GC cells and tissues (Bou Kheir et al., 2011). GC cells overexpressing miR-449 exhibit a senescent phenotype, as revealed by β-galactosidase assays. Luciferase studies also identified Geminin (GMNN), C-Met, Cyclin E2 (CCNE2), and SIRT1 as direct miR-449 targets. Additionally, miR-449 overexpression activated p53 and its downstream target p21, along with the apoptosis markers Caspase 3 and Poly (ADP-Ribose) Polymerase (PARP). These observations indicated that miR-449 induced senescence and apoptosis in GC cells by activating the p53 pathway (Bou Kheir et al., 2011).

#### 2.2.5 MiR-584-5p

MiR-584 is situated on 5q32 and is downregulated in GC cells (Zheng et al., 2017). Li et al. discovered that miR-584-5p inhibited GC cell proliferation and induced senescence in GC cells. Luciferase reporter gene assays identified WWP1 as a direct miR-584-5p target. Mechanistically, miR-584-5p promoted senescence in GC cells by downregulating WWP1 and activating TGF-β signaling, and implied that miR-584-5p may have therapeutic potential for human GC (Li Q. et al., 2017).

### 2.3 Colorectal cancer (CRC)

#### 2.3.1 MiR-34a

MiR-34a expression is strictly regulated by p53. Studies have indicated a mutual exclusivity between miR-34a methylation and p53 mutation in CRC, suggesting either miR-34a inactivation or p53 mutation in tissues or cells (Vogt et al., 2011). This implies that miR-34a may exert biological functions through the p53 pathway. Tsuchiya et al. reported that ectopic miR-34a expression significantly reduced colon cancer cell proliferation and induced a senescence-like phenotype. MiR-34a also participated in a positive feedback loop in the p53 tumor suppressor network (Tsuchiya and Nakagama, 2010).

#### 2.3.2 MiR-129-1-3p

MiR-129-1 is located on 7q32.1 and functions through both miR-129-1-5p and 3p (Ma et al., 2019). Fu et al. reported that significant miR-129-3p expression inhibited the E3 ubiquitin ligase Pirh2 and two other targets, IGF2BP3 and CDK6. MiR-129-1-3p, leading to the silencing of its target gene Pirh2, significantly increased p53 protein levels and its downstream target p21, subsequently inducing senescence in CRC cells (Fu et al., 2019).

#### 2.3.3 The miR-183/96/182 cluster

Positioned on 7q32.2, the miR-183/96/182 cluster is upregulated in CRC patients (Ahmed et al., 2013). The miR-183/96/182 cluster is composed of miR-183, miR-96 and miR-182, which are closely located in the chromosomal locus. Fu *et al.* identified an upstream regulator of the miR-183/96/182 cluster; avenanthramide C (AVN C) which induced senescence in CRC cells and reduced mature miR-183, -96, and -182 levels. Mechanistically, AVN C induced senescence by attenuating the β-catenin-mediated activation of the miR-183/96/182 cluster, thereby releasing their common targets: FOXO1, FOXO3, and SMAD4. This promoted p21 and p16 expression and indicated that the β-catenin/miR-183/96/182 cluster/FOXO1 axis induced senescence in CRC cells via the p21 pathway (Fu et al., 2022).

#### 2.3.4 miR-339-5p

Located on 7p22.3, miRNA-339 is significantly downregulated in CRC (Zhou et al., 2013; Zhou et al., 2015). MiRNA-339-5p directly inhibited MDM2 expression in CRC cells by binding to the MDM2 3′-untranslated region (UTR). As a key negative regulator of p53, MMD2 downregulation by miRNA-339-5p elevated p53 protein levels, enhanced p53 transcriptional activity, and induced cell apoptosis and senescence (Zhang C. et al., 2014).

#### 2.3.5 MiR-371b-5p

MiR-371b is part of the cluster with miR-371a, miR-372, and miR-373, and is regulated by cucurbitacin E (CE) as reported by Yang et al. (2022). Mechanistically, CE treatment increased miR-371b-5p, leading to significant TFAP4 inhibition. Silencing miR-371b-5p weakened p53, p21, and p16 expression by inhibiting TFAP4, thereby counteracting CE-induced senescence in HCT-116 CRC cells. This suggested that miR-371b-5p promoted senescence in CRC cells by inhibiting the TFAP4 protein (Yang et al., 2022).

#### 2.3.6 MiR-1827

Located at 12q23.1, miR-1827 is frequently downregulated in human CRC (Chen J. et al., 2023). MiR-1827 is a novel miRNA that targets MDM2 by binding to the 3′-UTR of MDM2 messenger RNA (mRNA). MiR-1827 negatively regulates MDM2, which in turn increases p53 protein levels to increase its transcriptional activity and enhance p53-mediated stress responses, including CRC apoptosis and senescence. Decreased miR-1827 expression was associated with high MDM2 expression and a poor prognosis in CRC. Thus, miR-1827 is a novel miRNA that regulates p53 by targeting MDM2 (Zhang et al., 2016).

### 2.4 HCC

#### 2.4.1 MiR-34a

Xu et al. identified a significant correlation between elevated miR-34a expression and advanced clinical–pathological parameters in HCC. Additionally, tumor tissues from 75 HCC patients showed negative associations between miR-34a levels and telomere indices (telomere length and telomerase activity). The transient transfection of HCC cell lines with miR-34a inhibited telomerase activity and telomere length, inducing a senescence-like phenotype and affecting cell viability. MiR-34a effectively targeted c-Myc and FoxM1, key players in telomerase reverse transcriptase transcription and crucial for maintaining telomerase activity and preventing senescence. Thus, miR-34a inhibited HCC by regulating telomere length during cell senescence (Xu et al., 2015).

#### 2.4.2 MiR-125b

Downregulated miR-125b expression in HCC samples was reported in several studies (Hua et al., 2019; Song et al., 2018; El-Naidany et al., 2022). Song *et al.* identified SIRT6 as the target of miR-125b in HCC in luciferase experiments. Subsequent cell studies demonstrated that miR-125b-induced functional changes could be rescued by restoring SIRT6. MiR-125b overexpression or SIRT6 knockout induced significant cellular senescence and apoptosis in HCC cells, aligning with the malignant transform of damaged HCC cells. Thus, miR-125b induced senescence in HCC cells by targeting SIRT6 (Song et al., 2018).

#### 2.4.3 MiR-145

MiR-145 is located on 5q32 and is downregulated in HCC tissues (Wang B. et al., 2019; Liang et al., 2018). Jia et al. investigated miR-145 expression in cancer stem cells (T3A-A3), BEL-7402 liver cancer cells, and normal liver sinusoidal endothelial cells. Lower miR-145 levels were observed in T3A-A3 cells. Upon miR-145 restoration in these cells, G1 cell cycle arrest occurred and was accompanied by a significant reduction in *in vitro* clone cell proliferation and *in vivo* xenograft tumor growth. MiR-145 restoration also curtailed tumor sphere growth *in vitro* and diminished tumor formation in nude mice injected with T3A-A3 cells. The tumor-suppressive impact of miR-145 in cancer stem cells was partially counteracted by Oct 4 overexpression, underscoring miR-145s pivotal role in HCC stem cell tumorigenicity via Oct 4 regulation (Jia et al., 2012).

#### 2.4.4 MiR-212

MiR-212 is located at 17p13.3 and is downregulated in HCC tissues (Jia et al., 2018). Liang et al. pinpointed H3K4 demethylase retinoblastoma-binding protein 2 (RBP2) as the target gene of miR-212 in luciferase reporter studies. Overexpressed miR-212 inhibited RBP2 expression, leading to suppressed cell proliferation and induced cellular senescence. Silencing RBP2 with short interfering (si)-RNA notably upregulated cyclin-dependent kinase inhibitors (CDKIs), curbing HCC cell proliferation, and promoting senescence. Thus, the miR-212-RBP2-CDKI pathway helped induce senescence in HCC cells (Liang et al., 2013).

#### 2.4.5 MiR-495

MiR-495 is located at 14q32.31 and is significantly downregulated in HCC tissues (Zhang et al., 2021; Ye et al., 2018). Its overexpression induced growth inhibition, G0/G1 cell cycle arrest, and a senescent phenotype in HCC cells. CTRP3 is a factor with pro-cancer and pro-inflammatory functions and was identified as a potential miR-495 target. Inhibiting CTRP3 expression with siRNA mirrored the growth inhibition observed with miR-495 overexpression. This suggested that miR-495 regulated HCC cell growth by targeting CTRP3, thereby modulating cell cycle progression and senescence (Zhang et al., 2021).

#### 2.4.6 MiR-4510

MiR-4510 is located at 15q14 and is downregulated in HCC tissues (Cartier et al., 2017). Ghousein et al. demonstrated that miR-4510 inhibited HCC cell proliferation and migration and induced a senescent phenotype by reducing RAF1 mRNA and protein expression. Functionally, miR-4510 interacted with RAF1 to directly target the RAS/RAF/MEK/ERK pathway, inactivating it and decreasing downstream target expression, such as c-Fos. As the RAS/RAF/MEK/ERK pathway is an acknowledged oncogenic pathway, miR-4510 may potentially induce cellular senescence in tumor cells via this pathway (Ghousein et al., 2020).

#### 2.4.7 MiR-139-5p

MiR-139-5p is located at 11q13.4 and is downregulated in HCC tissues. Wang et al. found the overexpression of miR-139-5p induced cellular senescence and inhibited proliferation of HCC cells. Then, the role of the EZH2/miR-139-5p/TOP2A axis in regulating cellular senescence and cell proliferation in HCC, enriched the molecular mechanisms of EZH2-mediated epigenetic regulation in HCC (Wang et al., 2023).

### 2.5 Pancreatic cancer

#### 2.5.1 The miR-17-92 cluster

The miR-17-92 cluster includes miR17, miR-19b1, miR-92a1, and miR-20a, which is located at 13q31.3 and is upregulated in pancreatic cancer (Huang et al., 2022a). Michele *et al.* reported that overexpressed miR-17-92 targeted several components of the NODAL/ACTIVIN/TGF-β1 signaling cascade, inhibiting downstream factors like p21, p57, and TBX3. This reduced self-renewal capacity, *in vivo* tumorigenicity, and chemoresistance in pancreatic cancer stem cells (Cioffi et al., 2015). Although the study did not explicitly investigate senescence phenotypes, p21 involvement–a key factor in the aging pathway–suggested the intriguing participation of ncRNAs in the interaction between pancreatic cancer stem cells and the p21 aging pathway.

## 3 LncRNAs

LncRNA studies have predominantly focused on CRC and HCC. While some lncRNAs are newly reported, the nine studies reviewed in this article have explored the intricate relationships between ncRNAs, senescence, and gastrointestinal cancers (Table 2).

**TABLE 2:** The characteristics of the lncRNAs participating in interactions between senescence and gastrointestinal cancers.

Model type	Species	NcRNAs	Expression	Biological functions	Mechanism	Reference
CRC	Human	LINC00858	Upregulated in CRC tissues and cell lines	Inhibited apoptosis, senescence, and autophagy in colon cancer cells	Promoted CRC progression via downregulating WNK2	Wu et al. (2020)
HCC	Human	LincNMR	Upregulated in HCC cell lines	Promoted proliferation and colony formation, inhibited senescence in liver cell lines	Regulated tumor cell proliferation through a YBX1-RRM2-TYMS-TK1 axis governing nucleotide metabolism	Gandhi et al. (2020)
CRC	Human	LncRNA VIM-AS1	Upregulated in CRC tissues	Promoted tumor cell proliferation, inhibited apoptosis, cellular senescence and arrested the cell cycle	Promotes tumor growth and metastasis by inducing EMT in CRC cells	Rezanejad Bardaji et al. (2018)
HCC	Human	LncR-PINT87aa	Upregulated in the hydrogen peroxide-induced HCC cell senescence model	Induced growth inhibition, cellular senescence, and decreased mitophagy	Binding to the DNA-binding domain of FOXM1, and affected the expression of FOXM1 itself but reduced that of its target genes involved in cell cycle and proliferation, especially PHB2, which was involved in mitophagy and transcribed by FOXM1	Xiang et al. (2021)
HCC	Human	LncRNA PANDA	Downregulated in HCC tissues	Promoted proliferation and carcinogenesis (nude mouse tumorigenicity) *in vitro* and *in vivo*	Repressed transcriptional activity of senescence associated inflammatory factor IL8	Peng et al. (2017)
CRC	Human	LncRNA PANDA	No difference	Low-dose curcumin could induce senescence in CRC cells without affecting cell apoptosis	PANDAR in curcumin-treated cells inhibited apoptosis and promoted senescence by stimulating the expression of PUMA	Chen et al. (2017)
HCC	Human	LncRNA PLK4	Downregulated in HCC tissues and cell lines	Induction of cellular senescence to inhibit liver cancer cell viability and growth	Talazoparib-induced lncRNA PLK4 could function as a tumor suppressor gene by Yes-associated protein (YAP) inactivation	Jia et al. (2020)
CRC	Human	LOC100507144	Upregulated in CRC tissues	Promoted apoptosis, cellular senescence, inhibited cell cycle, enhanced the migration and EMT, accompanied by the generation of cells with stemness characteristics	Enhance CRC progression and metastasis through regulation of the CD44/Nanog/Sox2/miR-302/miR-21 axis	Ebrahimi et al. (2022)
HCC	Human	LncRNA-miat	Upregulated in HCC tissues	Inhibited cellular senescence and promoted HCC progression	LncRNA-miat/miR-22-3p/sirt1 axis activated the tumor suppressor pathway (p53/p21 and p16/pRb) and stimulated senescent cancer cells to secrete SASP, which contributed to inhibition of tumor cell proliferation, and resulted in the suppression of HCC tumorigenesis	Zhao et al. (2019)
GC	Human	LncRNA SNHG6	Upregulated in GC tissues and in serum	Inhibited cell senescence	Promoted GC development by downregulating p21	Li et al. (2018)
GC	Human	LncRNA FGD5-AS1	Upregulated in GC tissues	Inhibited cell senescence	Through stabilizing YBX1	Qin et al. (2024)
HCC	Human	LncRNA NEAT1	Upregulated in HCC tissues and cells	Inhibited cell senescence	Via KIF11-dependent repression of CDKN2A	Chen et al. (2023b)
CRC	Human	LncRNA PCAT6	Upregulated in CRC	promoted cell senescence	NA	Han et al. (2024)

Note: GC, gastric cancer; CRC, colorectal cancer; HCC, hepatocellular cancer.

### 3.1 CRC

#### 3.1.1 LINC00858

LINC00858 is located at 10q23.1 and assumes a tumor-promoting role in CRC (Xu et al., 2020; Sha et al., 2019; Zhan et al., 2020). Wu et al. revealed that LINC00858 suppressed apoptosis, senescence, and autophagy in colon cancer cells, thereby propelling CRC progression via downregulated With-No-Lysine kinase 2 (WNK2) (Wu et al., 2020). Additional studies corroborated reciprocal targeting relationships between LINC00858 and WNK2 in CRC (Xu et al., 2020). However, WNK2 is a cytoplasmic serine-threonine kinase with pivotal roles in cell cycle progression, anti-apoptosis, invasion, and metastasis processes (Moniz and Jordan, 2010). Thus, LINC00858 may impede apoptosis and senescence thereby fostering CRC progression via its interaction with WNK2.

#### 3.1.2 VIM antisense RNA 1 (VIM-AS1)

VIM-AS1 is situated at 10p13 and promotes GC cell proliferation (Sun et al., 2020). Hajar et al. discovered that VIM-AS1 transcription was significantly increased in advanced lymph node metastasis and vascular invasive tumors. Downregulated VIM-AS1 arrested the CRC cell cycle at G2/M phase, induced apoptosis and cellular senescence, and impeded CRC cell proliferation (Rezanejad Bardaji et al., 2018).

#### 3.1.3 lncRNA-PANDAR

The promoter of CDKN1A antisense DNA damage-activated RNA (lncRNA-PANDAR) at 6p21.2 primarily regulates responses to DNA damage, is upregulated in CRC tissues (Lu et al., 2017), and is significantly associated with poor overall survival (Lu et al., 2017; Han et al., 2019; Li X. et al., 2017). During chemotherapy conditions, low-dose curcumin induced senescence in CRC cells without impacting apoptosis, while in curcumin-treated CRC cells, lncRNA-PANDAR expression increased. Silencing lncRNA-PANDAR in curcumin-treated cells enhanced apoptosis and potentially delayed senescence by stimulating p53 Upregulated Modulator of Apoptosis (PUMA) expression (Chen et al., 2017). However, PUMA acts as a checkpoint molecule limiting chromosomal instability in stem cells in response to telomere dysfunction. Therefore, lncRNA-PANDAR may delay CRC cell senescence via PUMA, which the latter is associated with telomeres and stem cells (antagonistic hallmarks).

#### 3.1.4 LOC100507144

Ebrahimi et al. discovered that LOC100507144 transcript expression was significantly elevated in tumors at advanced stages, with lymph node metastasis, and vascular invasion. Functional knockdown studies indicated that LOC100507144 propelled CRC cell proliferation by limiting apoptosis, inducing senescence, promoting cell cycle progression, enhancing migration, and inducing EMT. Moreover, LOC100507144 contributed to cell development via stem cell characteristics. Subsequent studies demonstrated that deleting LOC100507144 inhibited the expression of key stemness factors, including CD44, Nanog, and Sox2, thereby suppressing their targets; miR-302 and miR-21. These observations suggested that LOC100507144 induced CRC senescence and progression by regulating the CD44/Nanog/Sox2/miR-302/miR-21 axis associated with stem cell biomarkers (Ebrahimi et al., 2022).

### 3.2 HCC

#### 3.2.1 LncRNA PINT87aa

Xiang et al. reported lncRNA PINT87aa upregulation in senescent HCC cells. This lncRNA induced G1 phase cell cycle arrest and promoted HCC cell senescence by blocking the DNA-binding domain of the forkhead box M1 (FOXM1) protein and mediating the inhibition of prohibitin 2 (PHB2) transcription (Xiang et al., 2021).

#### 3.2.2 LincNMR

LincNMR is highly expressed in both liver and lung cancers and correlates with patient prognosis outcomes (Gandhi et al., 2020; Ota et al., 2023). Gandhi et al. observed G0/G1 phase arrest in HCC cells due to lincNMR loss and evaluated induced senescence. SA-β-GAL-positive blue cells indicated that lincNMR deletion triggered senescence in three liver cancer cell lines. Induced senescence promoted the expression of pro-inflammatory cytokines interleukin (IL)-1a, IL-1b, Endothelin (EDN), and Insulin Like Growth Factor Binding Protein 7 (IGFBP7), the former molecules being SASP factors, and the latter two being positive senescence-associated protein markers (Gandhi et al., 2020).

#### 3.2.3 LncRNA-PANDAR

Peng et al. reported that lncRNA PANDA overexpression promoted proliferation and carcinogenic effects in HCC both *in vitro* and *in vivo.* Mechanistically, PANDA inhibited the transcriptional activity of the senescence-associated inflammatory factor IL-8, thereby suppressing senescence in HCC cells (Peng et al., 2017).

#### 3.2.4 Polo-like kinase 4 associated lncRNA (lncRNA PLK4)

LncRNA PLK4 (GenBank accession number RP11-50D9.3) is a newly discovered lncRNA (Jia et al., 2020), the expression of which is significantly downregulated in HCC tissues and cells. Talazoparib is a novel and efficient poly ADP-ribose polymerase 1/2 (PARP1/2) inhibitor and increases lncRNA PLK4 expression in HepG2 cells. The authors reported that talazoparib-induced lncRNA PLK4 acted as a tumor suppressor by inducing YAP inactivation and cellular senescence and inhibiting cancer cell survival and growth (Jia et al., 2020). This observation suggested that lncRNA PLK4 may inhibit HCC progression by mediating YAP-induced cellular senescence.

#### 3.2.5 LncRNA-miat

Zhao *et al.* first reported that lncRNA-miat (myocardial infarction-associated transcript) was identified as an HCC-specific senescence-related lncRNA, highly expressed in HCC tissues and cells (Liu et al., 2020; Huang et al., 2018). Knocking out miat significantly promoted cellular senescence and inhibited HCC progression. Mechanistically, senescence-related lncRNA-miat acted as a competitive endogenous RNA to upregulate sirt1 expression by sequestering miR-22-3p. Additionally, lncRNA-miat downregulated the activation of some tumor-suppressive pathways (p53/p21 and p16/pRb) and stimulated SASP in senescent cancer cells to suppress tumor cell proliferation and inhibit HCC tumor occurrence (Zhao et al., 2019).

### 3.3 GC

#### 3.3.1 LncRNA FGD5-AS1

Qin et al. found a novel lncRNA named LncRNA FGD5-AS1 upregulated expressed in GC and inhibited the cell senescence via binding and stabilizing YBX1 which provided a novel strategy for the GC diagnosing and therapy (Qin et al., 2024).

## 4 CircRNAs

CircRNA exploration has been primarily confined to GC, CRC, and liver cancer, with many circRNAs still at early investigation stages (Table 3).

**TABLE 3:** The characteristics of the circRNAs participating in interactions between senescence and gastrointestinal cancers.

Model type	Species	NcRNAs	Expression	Biological functions	Mechanism	Reference
CRC	Human	Circ-MDM2	Upregulated correlated with mutated P53 samples in tissues	Decreased the MDM2 gene and P21 gene expression	Defected in G (1)-S progression via inhibiting the P53/P21 pathways	Chaudhary et al. (2020)
GC	Human	circGRAMD1B	Downregulated in GC tissues and cell lines	Inhibited the proliferation, migration, and invasion abilities of GC cells	Regulated miR-130a-3p-PTEN/p21 pathways involving in GC progression	Dai et al. (2019)
HCC	Human	circLARP4	Downregulated in HCC tissues and cell lines	Suppressed HCC cell proliferation, mediated cell cycle arrest and induced senescence	Regulated miR-761/RUNX3/p53/p21 signaling in HCC progression	Chen et al. (2019)
CRC	Human	circDNA2v	Upregulated in CRC tissues	Knockdown of circDNA2v induces high levels of senescence and proinflammatory SASP release	CircDNA2v functions through binding to IGF2BP3, preventing its ubiquitination, and prolonging the IGF2BP3 half-life, which in turn sustains mRNA levels of the protooncogene c-Myc	Wu et al. (2024)

Note: GC, gastric cancer; CRC, colorectal cancer; HCC, hepatocellular cancer.

### 4.1 Gastric cancer

#### 4.1.1 circGRAMD1B

Derived from GRAM domain-containing 1B (GRAMD1B), hsa_circ_0004798 is known as circGRAMD1B. In GC cells, circGRAMD1B acts as an miR-130a-3p sponge, restraining GC cell proliferation, migration, and invasion. Mechanistically, circGRAMD1B downregulated miR-130a-3p, subsequently upregulated Phosphatase and Tensin Homolog (PTEN) and p21 expression, and inhibited GC progression (Dai et al., 2019). The p21 protein pathway, a classical inducer of senescence, hinted at potential associations between this circRNA and senescence phenotypes.

### 4.2 Colon cancer

#### 4.2.1 Circ-MDM2

Derived from the MDM2 gene, hsa_circ_0027492 is known as circ-MDM2. Chaudhary et al. investigated correlations between p53 mutations and circ-MDM2, and reported higher circ-MDM2 levels in colon cancer samples with mutated p53. Inhibiting circ-MDM2 reduced MDM2 and p21 protein levels and caused G1/S phase cell cycle arrest in HCT116 colon cancer cells (Chaudhary et al., 2020). Although this study focused on cell cycle arrest, the downstream p53/p21 pathway suggested potential links with senescence effects.

#### 4.2.2 CircDNA2v

CircDNA2v was upregulated in CRC tissues. CircDNA2v functioned through binding to IGF2BP3, preventing its ubiquitination, and prolonging the IGF2BP3 half-life, which in turn sustained mRNA levels of the protooncogene c-Myc (Wu et al., 2024).

### 4.3 HCC

#### 4.3.1 CircLARP4

CircLARP4 is a circRNA derived from the LARP4 gene locus and is downregulated in ESCC, GC, non-small cell lung cancer (Zhang et al., 2017; Chen et al., 2020; Lu et al., 2020), and notably in HCC tissues and cell lines. Functional studies revealed that circLARP4 inhibited HCC cell proliferation, induced cell cycle arrest, and promoted cellular senescence. Overexpressed circLARP4 increased p53 and p21 levels, while silencing had the opposite effects. Mechanistic studies proposed that circLARP4, by targeting miR-761, inhibited HCC progression, elevated RUNX3 expression, and activated downstream p53/p21 signaling. These observations suggested circLARP4/miR-761/RUNX3/p53/p21 signaling involvement in HCC senescence and progression (Chen et al., 2019).

## 5 tRNAs

Transfer RNA (tRNA) primarily serves the purpose of transporting amino acids to ribosomes for the synthesis of proteins. However, recent research has revealed that specific tRNA fragments possess intrinsic biological functions. While the precise role of tRNA in gastrointestinal cancers remained to be fully understood, its potential association with the “senescence -gastrointestinal cancers” axis warranted further exploration. Guillon et al. observed that tRNA-Leu-CAA and tRNA-Tyr-GTA were upregulated during senescence escape, and their respective ligases, LARS and YARS, played important roles. Furthermore, it was found that mTOR inhibition prevented chemotherapy-induced senescence escape, accompanied by a decrease in the expression of tRNA-Leu-CAA and tRNA-Tyr-GTA (Guillon et al., 2021). Consequently, tRNA-Leu-CAA and tRNA-Tyr-GTA, along with their ligases, contributed significantly to the robustness and diversity of the tumor suppressive pathway.

## 6 Precancerous disease: Hepatic fibrosis

Some studies have suggested that intermediary diseases, such as chronic hepatitis and liver fibrosis, act as links between cell senescence and tumor development. For example, Karakousis et al. (2020) proposed that hepatitis B served as a connecting factor between cell senescence and HCC (Karakousis et al., 2020). Alcoholic hepatitis, liver fibrosis, and colorectal adenoma, recognized as precancerous conditions, served as valuable models when studying correlations between cell senescence and tumor onset and progression.

From alcoholic steatohepatitis progression to fibrosis/cirrhosis and eventually HCC, the underlying mechanisms remain unclear. However, Cen et al. indicated that c-Myc-regulated miR-17-92 contributed to increased p21 expression and oxidative stress during aging processes in adult mouse adipose-derived mesenchymal stem cells. This observation suggested the potential involvement of the miR-17-92 cluster in mesenchymal stem cell senescence via p21 signaling (Cen et al., 2023). Yang et al. also reported that the miR-145-ZEB2-p53 regulatory system may have triggered quiescent hepatic stellate cell (HSC) senescence, leading to liver fibrosis (Yang et al., 2019). Moreover, Wan *et al.* demonstrated a role for miR-34a in linking liver cell senescence and fibrosis. MiR-34a silencing enhanced HSC senescence, indicating that miR-34a promoted alcohol-induced liver fibrosis (Wan et al., 2017). In a bile duct-ligation mouse model, miR-34a, acting through Sirt-1-mediated aging and fibrotic responses, further emphasized this intricate relationship (Wan et al., 2023). The TGF-1/miR-34a/SIRT1 axis, as revealed in a primary sclerosing cholangitis model, demonstrated a SASP which contributed to biliary senescence and liver fibrosis via autocrine and paracrine mechanisms (Kyritsi et al., 2020). Modulation of the neutrophil SIRT1-C/transcription factor EBPα/miR-223 axis via senescence may have increased susceptibility to alcohol-induced liver injury, thereby showcasing miRNA roles in linking aging and alcoholic liver disease (Ren et al., 2022). Collectively, these studies suggested that miRNAs can act as intermediaries in mediating inflammatory responses in “senescence–gastrointestinal cancers” relationships.

## 7 Virus-sourced ncRNAs

In addition to encoded ncRNAs in the human genome, viruses also encode miRNAs. Wang *et al.* revealed that epstein-barr virus (EBV)-miR-BART3-3p promoted GC cell growth both *in vitro* and *in vivo*. BART3-3p inhibited GC cell senescence induced by an oncogene (RAS (G12V)) or chemotherapy (erlotinib). EBV-encoded LMP1 and EBNA3C, with known anti-aging effects, were expressed at low levels in epstein-barr virus associated gastric cancer (EBVaGC) specimens. BART3-3p inhibited cancer cell senescence in a mouse model and suppressed natural killer cell and macrophage infiltration into tumors by altering SASP. Mechanistically, BART3-3p directly targeted the tumor suppressor gene TP53 and downregulated the downstream target p21. The analysis of clinical EBVaGC samples also showed negative correlations between BART3-3p and TP53 expression. Current research has suggested that mutated oncogene RAS (G12V) or chemotherapeutic drugs also induced senescence. Wang et al. reported that RAS (G12V) and chemotherapy drugs induced BART3-3p expression in EBV-positive cancer cells, forming a feedback loop that maintained senescence in EBVaGC at low levels. Our results indicated that although TP53 was rarely mutated in EBVaGC, its expression was finely regulated. Therefore, EBV-encoded BART3-3p may have crucial roles inhibiting senescence in cancer cells (Wang J. et al., 2019).

## 8 Non-coding RNAs in extracellular vesicles

Extracellular vehicles (EVs), which are membrane-bound tiny particles released by cells, facilitate the transfer of information and substances between cells. In senescent cells, ncRNAs located around centromeres exhibit a notable open conformation and upregulate the expression of SASP-like inflammatory genes by disrupting the function of CTCF (CCCTC-binding factor). This disruption enhances chromatin accessibility and activates the transcription of inflammatory genes, ultimately promoting malignant transformation. Additionally, these ncRNAs can be transmitted to adjacent cells through small EVs and function as SASP-like inflammatory factors within the tumor microenvironment (Garcia-Martin et al., 2022). Yang et al. discovered that exosomal miRNA-146a-5p, produced by aged HCC cells, can inhibit HCC cell proliferation by suppressing aerobic glycolysis and promote HCC cell senescence by activating the CHK2/p53/p21 signaling pathway via targeting IRF7 (Yang et al., 2024). The ncRNAs present in extracellular vesicles play a crucial role in maintaining the balance between digestive tract tumors and aging.

## 9 Models

In this review, we identified ten reports which concentrated on senescence-related lncRNAs as prognostic markers in cancer (Table 4). These studies, predominantly in gastrointestinal and liver cancers, systematically analyzed differentially expressed lncRNAs between cancerous and non-cancerous tissues, which correlated with senescence-related genes, and facilitated predictive models for prognosis or survival time outcomes. Some models included risk score formulae, scoring tables or websites, and provided receiver operating characteristic curve predictions for 1, 3, and 5-year survival rates. Sensitivity analyses of the model and the association of the model with clinical parameters, immune microenvironment, or chemotherapy/immunotherapy were also conducted. Internal or external experimental validation data further solidified the findings (Huang et al., 2022a; Sun et al., 2022; Wang et al., 2022; Huang et al., 2022b; Gao et al., 2022; Zeng et al., 2022; Xu et al., 2022; Huang et al., 2023; Li and Xue, 2023; Zhang et al., 2019). Given the close associations between aging and cancer occurrence and development, lncRNAs differentially linked to gastrointestinal cancers and senescence genes may significantly impact tumor prognosis outcomes. Furthermore, studies examining senescence-related lncRNAs are more likely to identify markers relevant to immunotherapy, considering the profound connections between aging and immune responses. Importantly, these investigations have contributed valuable prognostic markers and potential therapeutic targets for assessing prognosis outcomes and implementing immunotherapy in patients with gastrointestinal cancers.

**TABLE 4:** Construction of a predict model using the cellular senescence-associated ncRNAs in gastrointestinal cancers.

Cancer type	NcRNAs	Model type	Model	Clinical features	ROC	Verification	Reference
HCC	LncRNA	Prognosis	Risk score = AC026412.3 × (1.6474) + AL451069.3 × (0.6620) + AL031985.3 × (1.0340)	Related with prognosis and immune	0.754(1-year), 0.675(3-year), 0.670(5-year)	qPCR in cell lines	Sun et al. (2022)
GC	LncRNA	Prognosis	Risk score = LINC02544 × 0.011000997 + LINC00571 × −0.813110635 + LINC00592 × 0.32778269 + POLMSH- AS1 × −0.025147195 + RRN3P2 × 0.221782955 + TYOS × −0.023627594 + LINC02696 × 0.860742118 + LINC01094 × 0.181390355 + LINC00449 × −0.027129427 + LINC01614 × 0.030126879 + PVT1 × −0.07100812	Related with age, sex, T stage, N stage, M stage, clinical stage, grade and the infiltration of the immune cells	0.714 (1-year in training group)	Internal verification	Wang et al. (2022)
HCC	LncRNA	Prognosis and the tumor microenvironment	Risk score = (0.6488 × AL117336.3 expression) + (−0.3616 × AC103760.1 expression) + (0.5888 × FOXD2-AS1 expression) + (−0.7890 × AC009283.1 expression) + (−0.3235 × AC026401.3 expression) + (−1.1729 × AC021491.4 expression) + (0.3475 × AC124067.4 expression) + (0.9231 × RHPN1-AS1 expression)	Related with HCC immunotherapy, chemotherapy, targeted therapy and TMB	0.774(1-year), 0.713(3-year), 0.793(5-year)	Internal verification	Huang et al. (2022b)
HCC	LncRNA	Prognosis and the tumor microenvironment	Risk score = 0.1483 × NRAV +0.1160 × AL139423.1 + 0.0950×AC008622.2 + 0.0513 × AL365203.2 + 0.0485 × LINC01138 + 0.0453 × AC009005.1 + 0.0326 × AC090192.2 + 0.0069 × SNHG3 + 0.0051 × AC026356.1 − 0.0094 × MIR100HG − 0.0355 × AC015908.3	Related with immune microenvironment and poor prognosis	NA	NA	Gao et al. (2022)
GC	LncRNA	Prognosis and the immunotherapy response	Risk score = (−0.0056 REPIN1-AS1) + (−0.0779 × AL355574.1) + (0.1341 AC104695.3) + (−0.5803 × AL033527.2) + (0.7814 AC083902.1) + (−0.1343 × TYMSOS) + (0.0129 LINC00460) + (0.0104 × AC005165.1) + (−0.6483 AL136115.1) + (−0.2790 × AC007405.2) + (0.8521 AL391152.1) + (0.0659 × SCAT1) + (0.7297 × AC129507.1) + (1.8695 × AL121748.1) + (−0.9227 × ADAMTTS9-AS1)	Related with Immunotherapy response	0.741(1-year), 0.819(3-year), 0.865(5-year)	NA	Zeng et al. (2022)
CRC	LncRNA	Prognosis and the tumor microenvironment	Risk score = (−0.8994×SNHG16 expression) + (−0.8211×AL590483.1 expression) + (0.9144×ZEB1- AS1 expression) + (0.6663×AC107375.1 expression) + (0.7681 × AC068580.3 expression) + (0.5576 × AC147067.1 expression)	Related with chemotherapy and targeted therapy	0.688(1-year), 0.718(3-year), 0.733(5-year)	Internal verification	Huang et al. (2022a)
CRC	LncRNA	Survival and tumor immune environment	AL590483.1, AC068580.3, AC147067.1, SNHG6, ZEB1-AS1 and AC107375.1	Related with immune microenvironment and drug sensitivity	0.771(1-year), 0.788(3-year), 0.758(5-year)	Internal verification	Xu et al. (2022)
HCC	LncRNA	Prognosis	AGAP11 and FAM182B	Related with immune escape and drug correlation	0.744(1-year), 0.718(3-year), 0.705(5-year)	The entire cohort	Huang et al. (2023)
HCC	LncRNA	Prognosis and Immunotherapy	Risk Score = (−0.025) ∗ MIR99AHG + (0.030) ∗ LINC01224 + (0.182) ∗ LINC01138 + (−0.326) ∗ SLC25A30AS1 + (−0.194) ∗ AC006369.2 + (−0.170) ∗ SOCS2AS1 + (0.204) ∗ LINC01063 + (−0.155) ∗ AC006037.2 + (0.256) ∗ USP2AS1 + (0.226) ∗ FGF14AS2 + (0.177) ∗ LINC01116 + (0.109) ∗ KIF25AS1 + (0.115) ∗ AC002511.2	Related with immune cell infiltration and immunotherapy response	0.8317(1-year), 0.8266(3-year), 0.8169(5-year)	NA	Li and Xue (2023)
GC	LncRNA	Prognosis	CeRNA network comprised of three lncRNAs (UCA1, HOTTIP, and HMGA1P4), 26 microRNAs (miRNAs) and 72 mRNAs	Cell cycle and senescence process	NA	HMGA1P4 by qRT-PCR	Zhang et al. (2019)
GC	MiRNA	Age-related STAD progression	MiR-124–3, miR-204, miR-125b-2 (Downregulated)	Related with Lauren type and tumor stage	NA	NA	Kim et al. (2015)

Note: ESCC, esophageal squamous cell cancer; GC, gastric cancer; CRC, colorectal cancer; HCC, hepatocellular cancer; NA, not available.

## 10 Future prospects

Currently, most research studies treat aging and cancer as independent phenomena. Frequently, following the exploration of ncRNA roles in aging, researchers transition to the cancer research realm, often neglecting to view the three elements (“ncRNAs–senescence–gastrointestinal cancers”) as an intricately interconnected triangular relationship. Those studies not exploring these factors as a triangular relationship frequently use cancer cells as a model for aging research. For instance, Xiang et al. reported lncRNA PINT87aa upregulation in senescent HCC cells, thereby promoting senescence (Xiang et al., 2021).

**FIGURE 2:**
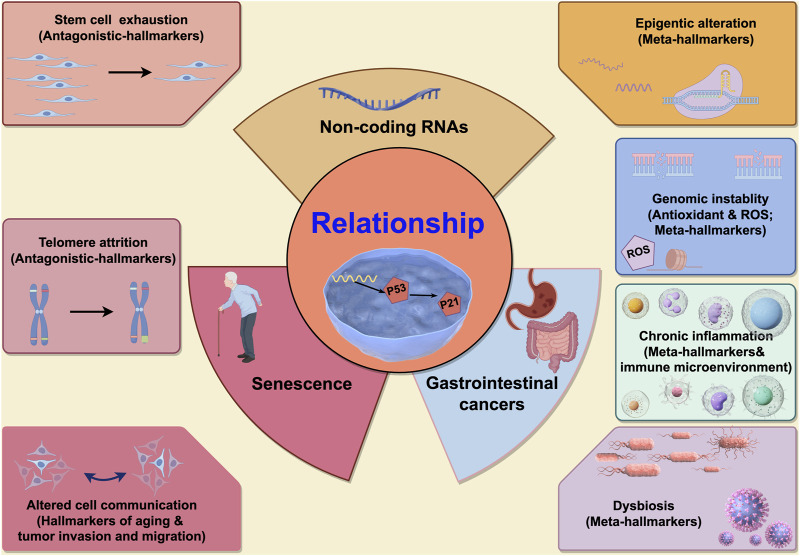
The characteristics of the non-coding RNAs participating in interactions between senescence and gastrointestinal cancers. Seven characteristics are participated in interactions between senescence and gastrointestinal cancers. In the left, two characteristics (stem cell exhaustion and telomere attrition) are antagonistic hallmarks between senescence and gastrointestinal cancers, and when ncRNAs participated in, the cell fate might be contradictory (means ncRNAs might promote the cell senescence and inhibit gastrointestinal cancers, or *vice versa*). In the right, four characteristics (epigenetic alteration, genomic instability, chronic inflammation and dysbiosis) are synergistic hallmarks between senescence and gastrointestinal cancers, and when ncRNAs participated in, the effect to cells might have cooperativity (means ncRNAs might promote the cell senescence and accelerate gastrointestinal cancers progression, or *vice versa*). And the last characteristic is altered cell communication which is only identified as one hallmark of aging (not as cancers), but this characteristic is associated with the gastrointestinal cancers (associated with the tumor invasion and migration). This picture is drawn used by the online software Figdraw (https://www.figdraw.com).

**FIGURE 3:**
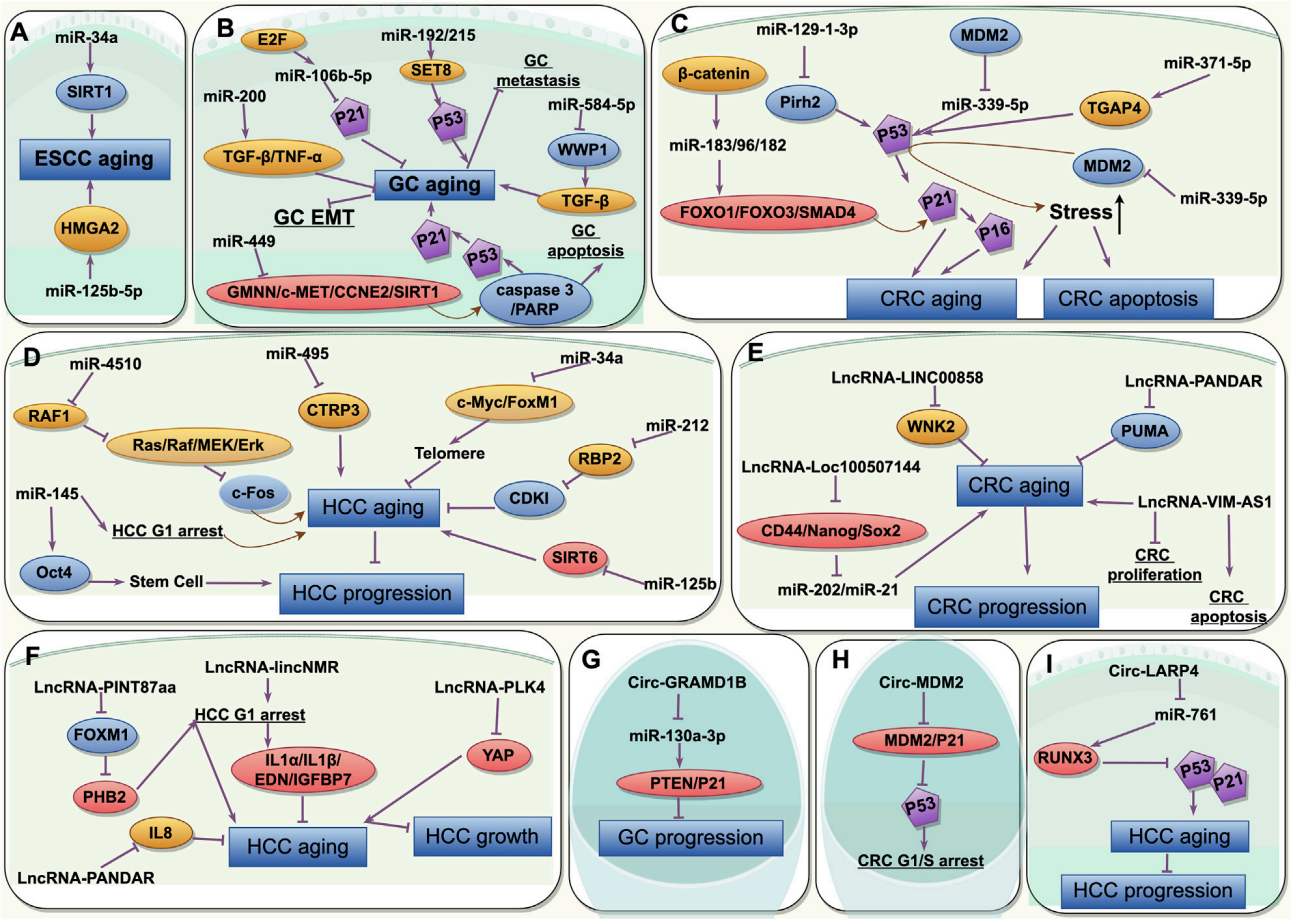
The common characteristics of the non-coding RNAs participating in interactions between senescence and gastrointestinal cancers and its mechanisms. **(A)** miRNAs in aging and ESCC; **(B)** miRNAs in aging and GC; **(C)** miRNAs in aging and CRC; **(D)** miRNAs in aging and HCC; **(E)** lncRNAs in aging and CRC; **(F)** lncRNAs in aging and HCC; **(G)** circRNAs in aging and GC; **(H)** circRNAs in aging and CRC; **(I)** circRNAs in aging and HCC. ESCC, esophageal squamous cell cancer; GC, gastric cancer; CRC, colorectal cancer; HCC, hepatocellular cancer. This picture is drawn used by the online software Figdraw (https://www.figdraw.com).

López-Otín et al. reviewed common meta-hallmarks shared between senescence and cancer, encompassing epigenetic alterations, with ncRNAs representing a mere fraction of this complex landscape. Critically, ncRNAs involved in senescence and gastrointestinal cancers interactions constitute multifaceted processes. Broadly, specific mechanisms fall into two categories: meta- and antagonistic-hallmarks (Figure 2). Meta-hallmarks encompass genomic instability (reactive oxygen species-induced oxidative DNA damage) (Maes et al., 2008), chromatin remodeling (Green et al., 2017), inflammation and the immune microenvironment (Cen et al., 2023; Yang et al., 2019; Wan et al., 2017; Wan et al., 2023; Kyritsi et al., 2020; Ren et al., 2022), the microbiome (Dalmasso et al., 2014; Huang et al., 2020), and changes in intercellular communications (including P53 and its downstream P21 pathway (Görg et al., 2018; Amaral et al., 2010), and TGF-β and WNT pathways (Ghafouri-Fard et al., 2021)). Antagonistic features include cancer stem-like cell depletion (Nautiyal et al., 2012) and telomere shortening (Vahidi and Samadani, 2021; Amano et al., 2019) (Figure 3).

NcRNAs have crucial relationship roles between “senescence and gastrointestinal cancers”. They regulate such relationships not only through their common mode of action and endogenous competitive inhibition, but also via multiple layers and perspectives, such as the genome, chromatin, stem cells, inflammatory mediators, and the microbiome, thereby forming an intricate network. Cell fate resembles a “carriage”, with the three main characters sequentially making appearances. These three factors may mutually propel cells toward both senescence and cancer via five meta-hallmarks, while two antagonistic features inhibit cells from progressing in both directions. However, cell fate toward cancer promotion or inhibition requires the consideration of organ or spatial-temporal phases and the magnitude of different driving forces. Whether ncRNAs promote or inhibit the development of these latter two factors, they not only act in isolation but rather interact on complex levels with other tumor features. And the limitation of this study is the articles included in the study limited only in English.

In terms of future research directions, a more direct examination of ncRNA roles in senescent gastrointestinal cancers cells and associated mechanistic studies is required. Research strategies may require senescent cells/animals for tumor modeling, detecting ncRNA involvement, or using tumor cells/animal tumor models for aging phenotypes to identify senescence-inducing ncRNAs and pathways. Additionally, comprehensive summaries/reviews for different cancer types and the exploration of network mechanisms are warranted. We believe that research related to tiRNA and piRNA will emerge in the near future. The future research directions would be focused on the potential of ncRNAs to serve as prognostic predictors or drug therapy targets for gastrointestinal cancers, or as innovative strategies to treat and manage gastrointestinal cancers in the elderly. When considering strategies for treating gastrointestinal tumors from the senescence perspective, we can focus on three key aspects: Firstly, by modulating the expression of specific non-coding RNAs (ncRNAs) and influencing their downstream potential target genes or pathways, we can induce senescence in gastrointestinal tumor cells, causing them to cease division and enter a stable state, thereby inhibiting tumor growth. Secondly, although senescent cells may produce factors that promote gastrointestinal tumor growth or metastasis, targeting these cells may mitigate their tumor-promoting effects. Thirdly, some downstream pathways of senescence-associated ncRNAs are linked to the immune system, suggesting that combining ncRNA modulation with immunotherapy may enhance the efficacy of both approaches (Afra et al., 2023). Consequently, by regulating the expression of these ncRNAs, we can impact the senescence process, tumor cell growth and metastasis, as well as immune system activity. Future research needs a more in-depth exploration of functions and mechanisms of ncRNAs to harness their potential more effectively in developing novel cancer treatment strategies. Additionally, it is crucial to address the potential side effects and safety concerns associated with ncRNA modulation to ensure their clinical safety and efficacy. Certainly, this field requires further in-depth investigations.

## Data Availability

The original contributions presented in the study are included in the article/supplementary material, further inquiries can be directed to the corresponding author.
